# Rigorous idiography: Exploring subjective and idiographic data with rigorous methods—The method of derangements

**DOI:** 10.3389/fpsyg.2022.1007685

**Published:** 2023-01-11

**Authors:** Chris Evans, Jo-anne Carlyle, Clara Paz

**Affiliations:** ^1^Escuela de Psicología y Educación, Universidad de Las Américas, Quito, Ecuador; ^2^School of Psychology, University of Roehampton, London, United Kingdom; ^3^PSYCTC.com, London, United Kingdom

**Keywords:** subjective data, psychometrics, qualitative data, idiographic data, derangements, statistical significance, psychotherapy research, rigorous idiography

## Abstract

Psychological research often seeks general rules applying across individuals, an aim that is in tension with examining that which is unique to any individual. There are general statistical regularities across individuals’ subjective self-report which enable much psychology and psychotherapy research to combine data from self-report questionnaire responses with statistical and psychometric methods to create a fundamental part of Cronbach and Meehl’s foundational nomological networks of validity. However, these methods only apply when most participants answer the same questions on measures creating nomothetic data and this has led to a neglect of idiographic data. This paper reviews a method of analysis of idiographic data, of “rigorous idiography”: the method of derangements. This is a remarkably simple statistical test of whether purely idiographic data convey reliable information. We show how the method appeared to become stuck in a bibliometric backwater but we expand on its potential for research and practise and hope it will be taken up and used correctly and more widely.

## Introduction

“… one cannot conceive of objectivity without subjectivity. Neither can exist without the other, nor can they be dichotomized. The separation of objectivity from subjectivity, the denial of the latter when analysing reality or acting upon it, is objectivism. On the other hand, the denial of objectivity in analysis or action, resulting in a subjectivism which leads to solipsistic positions, denies action itself by denying objective reality” (Freire, 1970, 50).

### Background

An examination and critique of the false binary of subjectivity and objectivity is long overdue which makes this edition of Frontiers in Psychology timely. This paper spells out the history of a novel method of testing idiographic descriptions: the method of “derangements.” We start by noting how the detection of regularities in answers to items in nomothetic questionnaires underlies the psychometric exploration of such measures and creates their important claims to be measures of subjective experiences: claims that turn on the regularities being statistically unlikely to have arisen by chance. These psychometric methods align with the desire for findings that are applicable and generalisable across people. However, this detection of regularities across people’s responses does not transmute the subjective into the objective, though it does weave subjective experience and statistical methods together constructively. Unfortunately, it is often presented as if self-report measures have validity across all respondents, as if the psychometric properties are independent of the respondents and are simply fixed qualities of each questionnaire. That presentation marginalises the subjective roots of the data and participants’ individuality. The dominance of these methods and nomothetic measures also marginalises exploration of purely idiographic data even though idiographic, subjective data *can* be tested for valid information content. The method presented here—derangements—shows that such idiographic information can be identifiable with better than random accuracy, the same test by which nomothetic questionnaire data can be shown to have its regularities of reliability and validity.

### Terminology

It is important to elucidate the use of three polarised terms in this paper: idiographic versus nomothetic; quantitative versus qualitative and objective versus subjective. These terms have somewhat different usages in different fields but the ways we use them in this paper are common in the psychological realm. We use “idiographic” for data which describes someone or something but is not a dimension on which everyone or everything has a numerical value, contrasting with “nomothetic” data drawn from such dimensions. For example, a picture of someone is idiographic data; their height and weight are nomothetic data. This use links with Millon’s argument that psychology needs both dimensional, i.e., nomothetic, measurements, but cannot do without descriptions of, and understandings of, individuals which Millon called “personology” (Millon, 2000). This use of these terms; idiographic and nomothetic, came into psychology through German philosophy and the work of Windelbrand (see translation Windelband, 1998). However, Windelbrand actually used nomothetic to describe the search for general rules and idiographic as the collating of individual historical facts.

We use “quantitative” simply for data involving numbers, as opposed to “qualitative” data which cannot be reduced to a simple dimension of numbers. In general, idiographic data is qualitative and nomothetic data is quantitative but this is not necessarily the case: a repertory grid with largely elicited elements and entirely elicited constructs is idiographic, i.e., unique to the individual who created it; however, the matrix of ratings given to each element for each construct is just a collection of numbers: quantitative data. Diagnostic and other categorisations may be qualitative: defined without numbers, but data from them become quantitative if they are counts of the categorisations.

Finally, we use “subjective” for that which comes from the mind of individuals and “objective” for that which seems external to minds: sadness is subjective and the length of a metre is objective. This is of course a philosophically vexed distinction but this somewhat pragmatic approach serves the distinctions being addressed in this paper.

### Subjectivity and style

As the opening quote from Freire underlines, we assert that the fields of psychology, therapy and humanitarian research all create unhelpful polarities between “objectivity” and “subjectivity” and in so doing marginalise both subjectivity and idiographic data. We suggest this marginalisation leans on a myth that psychometric methods distil objectivity from subjectivity, a myth that could be argued to dehumanise psychology and to evade the rich subjectivity at the heart of human research. This marginalisation has been also been inscribed into the literature in the expectations of a third person, passive, impersonal writing style (see Ping Alvin, 2014). So the central topic of this paper, of and the special issue: subjectivity extends to and invokes questions about grammar and writing stance and invites new thinking about exploration of subjectivity. Bearing this in mind, we have largely written the paper in the first person: bringing subjectivity to the foreground as this is congruent with the topic.

### Structure of the paper

We argue that the excellent logic underpinning psychometric methods honed for multi-item self-report questionnaires is often forgotten and that, instead of choosing methods and measures to fit the questions of interest, those methods and measures come to drive the questions that are asked. Perhaps surprisingly, we argue that a return to the logic of statistics and psychometrics offers valuable ways to test subjective, potentially purely idiographic, data using methods that can go some way to correct these false polarities. Throughout the paper our epistemological position is pragmatic, not claiming to show fundamental truths but suggesting methods whose value is in their utility.

We start by describing idiographic data, then we review the logic of statistical psychometrics arguing that, while it is excellent for comparisons of different individuals scores on nomothetic measures, it does not distil objectivity from subjectivity. Next we describe a method of “rigorous idiography,” specifically the “method of derangements” (Evans et al., 2002; Antonelli, 2019). Like nomothetic psychometric methods, this weaves together subjective and objective perspectives drawing on rigorous principles to subject entirely idiographic data to systematic statistical testing. We then show that this methodological approach has been known in the psychological literature for over 20 years but only generated one further use of the method despite its power and simplicity. Finally, we consider some of the reasons for this and juxtapose this with methods of classifying individual change on monothetic measures. Finally, we argue that the method of derangements is of value if new approaches to subjectivity are to be developed. We offer some potential applications, note the limitations of the method and propose avenues for extensions and further exploration.

## Idiographic data

All people draw on nomothetic data to communicate about each other: “she is very tall,” “she is mostly happy.” However, they also create idiographic descriptors which may contain nomothetic elements. Let us take a near cliché example from literature.

*Alas, poor Yorick! I knew him, Horatio: a fellow of infinite jest, of most excellent fancy: he hath borne me on his back a thousand times.*

Hamlet and Shakespeare are here using nomothetic scaling: “infinite jest,” “most excellent fancy.” The “infinite” nicely illustrates that the quantification is not to be taken too literally and “most excellent” is clearly ordinal but “jest” and “fancy” as used here are dimensions on which Hamlet might have rated anyone: nomothetic data. However, Shakespeare adds a purely idiographic personal element of history that locates Yorick as a person who has carried Hamlet on his back a thousand times: he is defining Yorick by a set of unique relational events. (They are counted but again the “thousand” is probably not to be taken as precise: technically quantitative, count data is building an individual picture.) Each individual who has ever lived is distinguishable from every other with enough of a picture or narrative: fingerprints, DNA, a photograph, even of a story of having been somewhere at a particular time: the first person to summit Everest is a very succinct narrative example.

Most humans build such word pictures of themselves and other people all the time, using dimensions of difference on various factors, nomothetic measures. For example, physical scales: age; height; number of chromosomes. We believe it is meaningful to identify some data as more objective than others: for example, age vs. feelings or personality traits. However, this is a somewhat slippery slope. For example age, apparently, a neutral, objective number, may carry multi-dimensional subjective meanings derived from both individual and social experiences and a 40th birthday may mean very different things to different people. This illustrates how objective versus subjective is not a simple dichotomy and that values on nomothetic scales can have individual meaning: being the *first* to summit Everest, not the 800th becomes an identifying, life defining and value.

Moving from the lay to the professional, there are many similar examples of psychological dimensions on which it can be assumed that everyone has a position but which are not purely “objective”: sibship position, level of education received, musical or mathematical capability or the seductively simple sounding idea of well-being. Although some of these appear to be based on observable, “objective” information, this is often not as simple as it seems. Take sibship position: this may conjure up an image of a particular idea of the boundaries of a family unit. Not only is this culturally laden, the question of how sibship can be consistently measured (for objectivity) is complex. For example, in counting sibship positions there may be differences in how the following are categorised: miscarriages, still-births, adopted and fostered siblings, half-siblings and step-siblings. These may seem like pedantic researchers’ concerns but to many people, they will feel important to their individual identity and how they see themselves located within family and community systems: crucial subjectivity and individuality.

Some psychological dimensions, like mathematical ability, are often measured in performance tests, so-called formative measurement, which may seem objective. However, formative measures can be based on cultural assumptions. Take mathematical ability: measures of this largely ignore different branches of mathematics and the complexity of the field. Let us shift scaling mathematical ability from nomothetic to idiographic. June Huh was one of the four 2022 winners of the Field’s medal, the Nobel Prize of mathematics, surely one end of a nomothetic, if complex, scaling of mathematical ability. At school, Huh performed poorly on school tests of mathematical ability and dropped out of secondary school to write poetry before later finding a new interest in mathematics, at which point, when applying for a PhD, he was rejected by every university in the United States but one: even performance measures can hide uniqueness and the subjectivity of deep and complex abilities (Cepelewicz, 2022; Editorial, 2022).

As the example of June Huh’s story shows, idiographic descriptors subsume data which may be both quantitative and qualitative but is built into a narrative data of sufficient “thickness” that it is located in an individual. Such data does not have to be textual, it could be photographs of faces, pieces of music or film of movement. In psychology, such data generally originate from an individual. That individual does not have to be identifiable: one of the great ethical challenges of narrative case reporting of therapy case data is to have data that is accurate, honest, conveying the elements of the individual that are of interest, but at the same time the data should be such that no-one could identify that person (assuming that was the undertaking that had been given). There is another area of psychology in which the idiographic data could be non-human, typically in studying perception and emotional or other reactions. Such work can use photographs of different trees to explore people’s reactions to nature, or recorded noises to explore attitudes to environmental context; if the data are complex enough to be unique to a particular tree or unique source of sound, they are idiographic data. Such data can also be virtual not real: virtual reality rooms, avatars and game scenarios could all be complex enough to be unique.

In the realm of the psychological therapies case reports, extended interview quotes, explanations of how someone did something can all reach sufficient “thickness” to be idiographic: uniquely describing one person and not simply aggregating a combination of outcomes on multiple nomothetic dimensions on which everyone can have a score. Such idiographic data have a long history and could include a therapist’s formulation of a client’s presenting problems, “self-characterisation narratives” (Kelly, 1955; Androutsopoulou, 2001); any transcript of part of a therapy session (e.g., Avdi and Evans, 2020); Core Conflictual Relationship theme analyses (Luborsky et al., 1994); Self States Sequential Diagrams (Bennett and Parry, 1998) and reformulation letters in Cognitive Analytic Therapy (Ryle, 1995), or music or art created in arts therapies. Unlike the items in a nomothetic questionnaire, no single part of such data can be expected to map to a similar descriptor from or about another person. The first sentence of one person’s self-characterisation is not directly numerically comparable with the first sentence of another person’s. Similarly, the tenth speech turn of a therapy transcript cannot be measured against the tenth turn from another session of the same patient and a formulation of one client may have no obvious points of comparison with that of another client.

Some such data have traditionally been used as the basis for nomothetic categorisations or ratings, in effect turning idiographic data back to nomothetic. Examples include indices created from idiographic repertory grids (*inter alia* Bieri, 1955; García-Mieres et al., 2019) for “grid complexity” and (Paz et al., 2019, 2020a) for dilemmatic cognitions. The application of the word “test” to repertory grids (Bannister et al., 1971), to coding of Rorschach (Wellington and Wellington, 2022) and House-Tree-Person images (Vass, 1998) and the application of the Formal Elements Art Therapy Scale (Gantt and Anderson, 2011) to images created in art therapy are all examples of conversion of idiographic data to nomothetic scaling or categorisation. However, none of those coding systems could use the dominant methods of psychometrics which we come to next.

## Psychometrics of nomothetic, multi-item measures: Distillation of subjective data to scores

In the psychological realm, attention is often focused on dimensions that are clearly subjective but where it may be useful to assume all individuals have a value at any point in time: classically nomothetic data. These dimensions, such as depression, anxiety, or obsessionality, lie at least partly in the psychiatric and mental health arenas, and have been key areas of measurement for psychological researchers. Over the last century measurement of such dimensions has been dominated by use of multi-item, self-report questionnaires and “standardised” or “semi-standardised” interviews (see Hamilton, 1967; Wing et al., 1974; First et al., 1997 for varyingly standardised interview schedules). These measures, with allied psychometric and statistical procedures, have almost eclipsed other measurement approaches and created the myth that mathematical distillation of item scores create “objective” data, ignoring the reality that these scores were derived from what can only ever begin as subjective response choices.

The field of psychometrics has been split to a large extent between “classical test theory” (CTT) and “item response theory” (IRT). Although these methods start from different sets of assumptions, they each assess, in any sample of responses on questionnaires, that there are regularities in those responses and that these regularities reveal similarities and differences among the respondents, allowing the psychometric analyses to locate the respondents on the dimension the instrument aims to measure. The mathematical methods, whether CTT or IRT, show that there is only a small possibility that differences in the item scores for the participants are random, thus supporting the idea that some dimensions of difference are being distilled out from random noise in the data. These methods, though originating with qualitative and subjective ratings, create structures to ascribe reliability and validity to these data (see Cappelleri et al. (2014) for an overview of both CTT and IRT and one with a very clear acknowledgement that validity starts with qualitative data).

It is important to note that such methods cannot reveal what was going on inside the mind of any person responding on the measure. These methods presume some shared similar processes and perceptions across participants completing the measures while acknowledging there will also be some differences between people. Ultimately subjectivity drives the responses that people give and may impinge on different items differently, for example:

“Although a well-constructed questionnaire will try to minimise individual differences in interpreting particular items, people always bring their own frames of reference to the task and their interpretations of items may vary considerably. For example, if an item says ‘I have felt warmth or affection for someone’, how people understand ‘warmth or affection’ will differ to some extent between individuals. A person who has not had much warmth or affection in their life may answer ‘sometimes’ if they regularly chat to the person in their local corner shop” (Evans and Carlyle, 2021, chapter 2, p. 29).

That sort of personal history may impinge on interpersonal items in the measure but not on more purely intrapersonal items. In that book (Evans and Carlyle, 2021) we strongly support the value of these psychometric methods, rooted in objective mathematical and statistical procedures, and we argue for wider use of measures in psychological therapies. However, there, and in Paz et al. (2020b), we also argue that for these methods of measuring the subjective to remain relevant and authentic, it is essential to avoid equating such questionnaire data with physical measures like height, weight and blood glucose. Just as scores are reported converting subjectivity to objectivity, the regularities across samples are almost always reported as if they define fixed qualities of the instruments rather than of the sample under consideration and, particularly worryingly, as if these fixed qualities apply in the same way for everyone using the instrument. That this cannot be the case can be demonstrated by *reductio ad absurdum*: if you give a self-report questionnaire to someone who cannot read it does not matter how good the reliability of the measure is, that individual’s answers will be random or absent. To take a less extreme example, someone entering adulthood will have different perceptions than someone facing the end of life and these may affect how each answers questions about quality of life and about activities of daily living. Questionnaires can almost never have fixed properties that are constant across the people who are answering them in the way that a weighing scale may have stable validity across a range of people standing on it. Individual intentions can impinge: almost always if someone wants to misrepresent their internal state they can. Self-report measures simply do not have fixed measurement properties that apply for everyone for all that they can have regularities across many people.

These regularities in sample data and, by generalisation, population data, indicate to what extent differences between individuals’ scores on measures should be seen as indicating minor differences or substantively important ones: this is what these questionnaire and interview measures are for. Some methods, e.g., Cronbach’s alpha (Cronbach, 1951) as an index of “internal reliability,” or “internal consistency,” largely ignore dimensionality of the systemic variance in the data, while others, typically factor analysis, attempt to partition that systematic variance into defined separate dimensions. The latter methods may show well-being to have separable dimensions that might include anxiety and depression as well as an overall dimension of well-being.

Psychometric methods are generally applied to single completions of measures and partition the item scoring differences between respondents into shared and random variance, i.e., treating individual differences in patterns of responses as “random.” Even when there are repeated completions by individuals, the maths of the methods necessarily restrict individual differences to be differences on shared dimensions of variance, not idiosyncratic differences: individual differences in change become evidence of failed “longitudinal measurement invariance” (e.g., Rosenström et al., 2022) or of shared “response shifts” (Murray et al., 2020). However, see Beurs et al. (2015); and Fried et al. (2016) for views that do not expect strongly shared patterns of responding. Molenaar (2004), in a polemical paper, showed mathematically that even if a general psychometric structure may be found in cross-sectional data, there is no reason that the same structure will be found in repeated data, or even that the individuals who show the shared cross-sectional regularities in their responses will all show the same longitudinal patterns.

Quantitative exploration of the limits of psychometric generalisability are themselves fairly limited despite a long history. Work has shown clearly that individuals differ in temporal stability on many variables (Epstein, 1979, 1983; Cooper and McConville, 1990; McConville and Cooper, 1997). How measures are presented affects responding (Braho and Bodinaku, 2015) and it is known that there is typically a mean shift in scores when mental health or well-being measures are completed twice by non-help-seeking samples (Durham et al., 2002). There is also a growing literature meta-analysing psychometric properties starting with the issue of “reliability generalisation” (Vacha-Haase, 1998; Deng et al., 2019). For almost all measures meta-analysed in this way, the results show significant and sometimes larger differences in psychometric properties across samples.

These quantitative analyses show that though there are generalities when people complete nomothetic questionnaires, the processes behind individuals’ item responses are not as simple as it might seem. There is also a qualitative literature on this (e.g., Blount et al., 2002; Kelly et al., 2012; Truijens et al., 2019; De Smet et al., 2020) and this shows marked diversity across individuals’ views of measures, and in their views of their own change. The qualitative, idiographic, descriptions, can be substantially different and more complex than that shown by simple score changes. The sophisticated psychometric tools of CTT and IRT help to explore regularities in item scores for whole samples, but they do not distil objectivity from subjectivity: they extract regularities across subjective responses.

None of this undermines the core utility of CTT or ITT, these are good tools that give general simplifications of often complex and, when brought to psychotherapies, often very personal issues. The limits of the methods and the tools are fairly clear as are the dangers. McLeod (2001) drew attention to the sociological and epistemological problems of overvaluing and dehumanising questionnaire data, terming the result “an administratively created reality.”

To summarise the argument so far: psychometric methods that clarify regularities in the subjective answers to self-report measures are logical and valuable but often overvalued. These methods require regularities in subjective appraisal across multiple individuals, and multi-item nomothetic measures depend for their “validation” on these psychometric methods by assuming that everyone answering the measure is asked the same questions. This creates a circularity: that the methods rely on all respondents being asked the same questions and then, that only measures that have been deemed to have been validated by those methods are accepted as “scientific.” This has contributed to a neglect of individual, personal, subjective experience where there can be no regularities across individuals and it has sidelined idiographic data and experience. The very real strengths of these methods have sadly created two mythological by-products: firstly that the methods convert subjectivity to objectivity and secondly that they reveal fixed measurement properties of the measures *that apply for everyone using them*. Challenging both myths is clearly at the heart of this special issue of the journal.

In the next section we return to the question of what idiographic data is, then in Section 3, we show how the method of derangements allows rigorous exploration of idiographic information, objective or subjective, and can help redress the neglect of what is importantly individual, and can also support the interweaving of the subjective and the objective.

## Rigorous idiography

As noted above, examples of idiographic subjective data in psychology are myriad. Over the last half century, there has been interest in such data but it has been almost entirely designated as “qualitative data” and analysed by an increasingly well-developed set of qualitative analytic methods (e.g., Smith, 1995; Braun and Clarke, 2006, 2021; Stapley et al., 2022).

Despite the developments in qualitative methods, there remains a very clear frontier between quantitative and qualitative methodological realms, with quantitative data generally being given higher prestige or being seen as more “sound” (e.g., Galasiński, 2021). Qualitative data and findings are generally designated “soft,” and questionnaire scores as “harder” than qualitative data, despite the fact that both start from fundamentally subjective experiences. As shown in the last section, this attribution of “hardness” to data rests on the application of maths, of psychometric, statistical methods to individuals’ subjective responses to questionnaire items. The maths is used to partition variance between random and systematic data, dignifying the systematic variance as “hard.” However, it is perfectly possible to apply the mathematics of statistics and randomness to decide if there is something systematic in idiographic data. The approach we illustrate here is this “method of derangements” (Evans et al., 2002).

### Method of derangements

This method is based on the idea of using a judge to match descriptors to their source: for example, can a therapist who worked with four different clients match unique self-characterisation narratives from the clients, on the basis of her work with the clients, despite never having seen the narratives?

The method needs at least four sets of data and some separate information about the source of these data. The data could be “House-Tree-Person” (Buck, 1948; Buck, 1966) drawings by six clients and the task to match the drawings to psychoanalytic formulations of the same clients’ difficulties created by therapists who never saw the drawings. Equally, the data could be 10 transcripts of “rupture/repair” sequences in therapy sessions and elicited repertory grids from those 10 clients: could a judge map the transcripts to the grids?

The probability of matching four or more objects correctly is unlikely to happen by chance alone. Remarkably, it will happen by chance with *p* < 0.05 regardless of the number of objects. A judge mapping all four objects correctly achieves something possible by chance alone with probability of 1 in 24. Why 1/24? Because there are 24 ways of rearranging four objects: four ways of picking the first, three ways of picking the next from the remainder and two ways of picking the penultimate one: 4x3x2 = 24. There is, of course, only one way of picking the last one after picking the first three. Equally obviously, only one of those 24 mappings is the correct one so the chances of achieving a correct mapping of all four is 1/24, and this is below the conventional criterion of “*p* < 0.05” for “statistical significance.” What is interesting, perhaps counter-intuitive but mathematically true, is that to match four or more correctly from *any* number of descriptors has a chance probability of *p* < 0.05, which we will demonstrate below.

The steps of the matching task are shown in Table 1 and the task is always for a judge (or judges) to map the data to their sources. To take the first example in the opening of this section—matching self-characterisation narratives—the challenge is for the therapist to match the self-characterisation narratives to the individuals who created them. This design involving mapping data to the people from which it came is only one configuration; the challenge could be to match two different idiographic descriptors, for example, assessment formulations and Rorschach reactions and then the judge does not have to know the individuals behind those data. The data do not have to be personal: in medical training, the challenge could be to match a set of blood biochemistry profiles to a set of diagnoses; in horticulture, it could be matching photographs of plants to the species names and in oenophily, it could be matching blind tasted wines to their origins; or—as can be seen below for mathematics—it might be to match a set of equations to graphical plots of those equations. However, the great strength of the method for psychology and the exploration of subjectivity is that it can be applied to entirely idiographic, purely personal, data: it needs no regularities between individuals.

**Table 1:** The stages of the method of derangements.

1. Assemble four or more idiographic descriptors and a separate identifier, e.g., repertory grid data and IDs of clients who completed the grids.
2. Randomise the order in which you will present them to a rater of one set of descriptors (allowing that this may randomise them into the correct order).
3. A rater who knows the clients but not the idiographic descriptors, i.e., has never seen the clients’ grids attempts to match the descriptors to the clients.
4. Look up the cumulative probability that the rater would have scored a matching score at least as high as they did and compare with your predetermined criterion to decide whether or not to reject the null hypothesis that there is no non-random transfer of information from the descriptors to the rater allowing them to match descriptors to their origin. Lookup tables can be found in Table 2 or at https://link.psyctc.org/derangements

We will set out two examples of how the method has been used to give a more meaningful sense of its potential applications. In the original paper about the method (Evans et al., 2002), the data were plots from repertory grids completed by six members of a forensic psychotherapy group and therapists from the group, who had not previously seen the grids or plots, were asked to match the plots to the patients. In Walters (2005) study, the idiographic descriptors were Cognitive Map of Major Belief Systems (CMMBS) maps for 19 clients from three drug treatment groups, and the rater was the therapist from the groups. The method is equally applicable to descriptors from the same individual at different times. For example, the descriptors could be self-characterisations from one client prior to therapy and after 10, 20, 30, 40 and 50 sessions and 6 months after termination. Similarly, they could be self-characterisation drawings from one woman in each of three trimesters of pregnancy and then at three monthly intervals for 2 years post-partum. Clearly, for the method to be giving important information, whatever the data, it is important that spurious identifying information be removed: for example, the actual names of people forming the elements of the repertory grids must be removed. Similarly, very unusual words used by an individual in a self-characterisation and appearing in that individual’s therapy transcripts would need to be removed. As ever, a method is only as good as the careful, logical, thoughtful use made of it.

It may seem counterintuitive that the criterion for statistical significance remains the same regardless of the number of descriptors, but this happens because the number of possible wrong mappings goes up very rapidly as the number of descriptors goes up, thus keeping the criterion for *p* < 0.05 to four. This is shown in Table 2 which shows all possible scores for four, five or six descriptors. There are, of course, 24 possible ways to arrange four objects, 120 ways for five objects and 720 ways for six objects. For scores for up to 30 descriptors see https://link.psyctc.org/derangements. If the judge correctly matches at least four of the descriptors this always has a likelihood of happening by chance alone of less than one in 20, i.e., meeting the classical criterion for statistical significance (and thus rejecting the null hypothesis). As can be seen in Table 2, this applies to: four out of four (*p* = 1/24); four or more of six (*p* = 0.022); four or more of seven (*p* = 0.018). Four of five descriptors is impossible: if you have mapped four correctly the remaining one can only be matched correctly, the probability of five of five is one in 120, i.e., *p* = 0.0083.

**Table 2:** Possible scores for four, five, or six descriptors.

Number of descriptors	Score (number matched correctly)	Number of ways of getting that score	Point probability of score	Cumulative probability of a score as high or higher
4	4	1	0.0417	0.0417
4	3	0	0	0.0417
4	2	6	0.25	0.292
4	1	8	0.33	0.625
4	0	9	0.375	1
5	5	1	0.00833	0.00833
5	4	0	0	0.00833
5	3	10	0.083	0.0917
5	2	20	0.167	0.258
5	1	45	0.375	0.633
5	0	44	0.367	1
6	6	1	0.00139	0.00139
6	5	0	0	0.00139
6	4	15	0.0208	0.022
6	3	40	0.055	0.077
6	2	135	0.188	0.265
6	1	264	0.367	0.632
6	0	265	0.368	1

A graphic summary of the method is given in https://www.psyctc.org/psyctc/2022/07/23/sometimes-n4-is-enough/.

### History and impact of the method of derangements

This method turns on mathematics that has been known for centuries. However, the first description of the use of the method in psychology was published by Evans and colleagues 20 years ago in a high-status journal specialising in the mathematical and statistical areas of psychology (Evans et al., 2002). Despite this respectability, a recent search found only four citations of the paper. The relatively niche nature of the journal in which it was published may have contributed to the low impact of the paper, however another potentially limiting factor is that the method was described for a mathematically inclined readership, perhaps demonstrated by the fact that the process of the method was clearly misunderstood by one of the publications citing it.

The use of the method in two forensic papers may be linked to the fact that two of the original authors were known in forensic research circles or to the reality that serious offending is very personal and idiosyncratic, which may create a willingness to focus on the subjective and the idiographic more than is the case in the more general areas of psychology and psychotherapies. Nonetheless, that there is some diversity of applications even in these four citations suggests the potential usability of the method in a variety of contexts. We now summarise the papers that have cited the original paper to explore the reasons for its limited uptake (Table 3).

**Table 3:** Description of papers citing the method of derangements.

Author(s)	Year	Title	Journal	Notes
Walters	2005	Mapping the criminal mind: Idiographic assessment of criminal belief systems	International Journal of Offender Therapy and Comparative Criminology	Uses method correctly
Daffern, Howells, Mannion and Tonkin	2009	A test of methodology intended to assist detection of aggressive offence paralleling behaviour within secure settings	Legal and Criminological Psychology	Method used was actually not the method of derangements and so criterion for *p* < 0.05 did not apply.
Sales and Wakker	2009	The metric-frequency measure of similarity for ill-structured data sets, with an application to family therapy	British Journal of Mathematical and Statistical Psychology	Tangential. Does not use the method.
Antonelli	2019	A surprising link between integer partitions and Euler’s number e	The American Mathematical Monthly	Arose from setting an exam question in which students were asked to map nine equations to nine plots. Develops the mathematical theory using different methods from that in Evans et al. (2002) paper but confirming the finding

Walters (2005) used the method correctly, reporting that 19 of 19 offender clients in a drug treatment programme were matched correctly to their idiographic self-description maps (Walters, 2005, 18). Usefully the therapist who did the matching was asked what aspects of the self-descriptions were most helpful and she gave explanations for what cues she had used. The paper has been cited twice but neither publication uses the method of derangements.

Sales and Wakker (2009) cite the derangements paper but report the mathematical logic behind another method, the “metric-frequency (M-F) measure.” This indexes the similarity between individuals where there are ratings of the individuals with at least some overlaps in the ratings used for each person. The M-F method is undoubtedly another rigorous method for analysing subjective and at least partly idiographic data and is an excellent method to create an index of similarity of personal questionnaire self-descriptions from separate respondents. However, the similarity indices have no probabilistic interpretations: high similarities could occur “by chance” and what “chance” similarity would mean in terms of the index is hard to define. The authors explain that the method of derangements and the M-F method address only distantly related issues and are mainly linked by the desire to demonstrate how mathematical concepts can be used to meaningfully help in the management of idiographic data.

Daffern et al. (2009) conducted a very interesting study investigating “offence paralleling incidents” in a forensic setting. They wished to test the idea that untoward incidents in treatment often show similarities with the patient’s original offences which might be the case if both the offences and the incidents are being driven by the same, perhaps highly individual, internal dynamics. For example, it might be that one offender killed someone reacting to a very particular sense of shame involving an older adult looking down on them in an interaction with a peer, perhaps echoing a childhood experience. This could recur in the inpatient setting where the person might react violently in a similar constellation of events. Daffern et al. had data on 97 incidents caused by 31 patients in a high secure forensic treatment unit, collating 86 nomothetic descriptors of index offences and incidents. The method of derangements was not actually used as the authors deemed there to have been a match if at least four of the 86 descriptors were the same for the index offences and incidents. This is a similarity count not a matching task. Sixty of the 97 incidents were deemed to match that patient’s index offence by this criterion but this could very easily have happened by chance alone with so many descriptors and their base rates (the frequency with which they are used across the sample) being fairly high. The rating used is more similar to the M-F method than to the method of derangements.

The method of derangements could have been used for this data set. For example, raters might have been given descriptions of the index offences of each of the 26 patients and descriptions of the patients’ inpatient incidents, one per patient (selected at random from each patient’s incidents). That then would have been a matching task and four or more correct matches across the 26 would have been unlikely to have happened by chance alone (*p* = 0.019). The paper has been cited 11 times but none of the citing works claim to be using the method of derangements.

**Figure 1:**
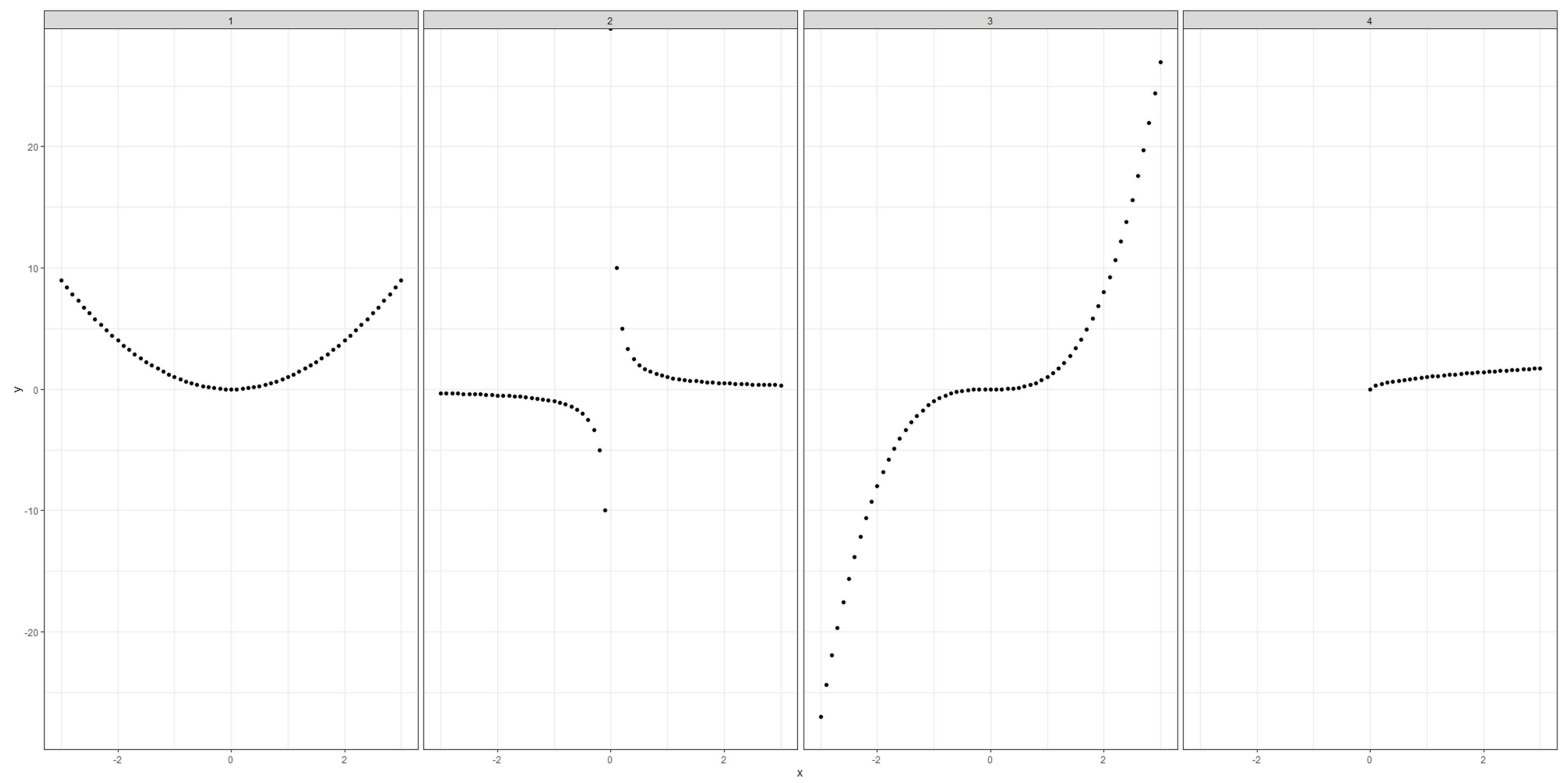
Equation plots to demonstrate the use of the method of derangements in Antonelli (2019).

Finally, Antonelli (2019) offers a fascinating extension of the mathematical theory behind the derangements method, as well as a clear application of the method, illustrating how it can be used for objective data as well as subjective data. The author wrote the paper after setting a maths exam question asking students to match nine equations with nine plots. For a simple example, consider only four equations: *y* = *x*^2^, *y* = 1/*x*, *y* = *x*^3^, *y* = *x*^0.5^ and the following plots (Figure 1).

After setting the exam Antonelli wondered about the probabilities of the students getting nine out of nine correct. He gives two separate and sophisticated mathematical methods, different from those in Evans et al. (2002), exploring the probabilities. All three methods of course give the same results. Antonelli comments:

“The probability that a student guesses all n correctly is *p*_X_ (*n*; *n*) = 1/*n*!, since there is only one correct permutation out of the n! possible. For the motivating example with *n* = 9, a student is more likely to be struck by lightning during the next 3 years than guess all correctly! The probability of getting exactly *n* − 1 correct is *p*_X_ (*n − 1*; *n*) = 0, since if a student gets *n* − 1 correct, the remaining choice must form a match” (Antonelli, 2019, p. 423).

This a mathematical paper beyond our grasp of maths and has not been cited yet. However, Antonelli alerted us to the fact that the same mathematical procedures of the derangements method have been used in devising an optimal way to allow a swarm of minimally computationally powerful drones to map and classify objects in their environment (Majcherczyk et al., 2021). Perhaps, humans own sensory neural systems use similar methods! We noted that the relaxed mix of subjectivity and maths in the writing style of those two papers is unlike that of much quantitative psychology writing where the subjectivity of the authors is generally hidden. It is interesting to note that the question of whether maths is objective or subjective is still an open discussion in work on the philosophy of maths.

## Discussion

One reason why subjective, sometimes purely idiographic, psychological data is neglected is that it is seen as not having the infrastructure of “hard” mathematical, statistical, psychometric methods that are the norm for analyses of nomothetic data. As we have argued above, the assumptions that surround nomothetic data tend to hide the fact that most psychological data on nomothetic questionnaires is subjective and remains subjective even when psychometric tools are used to show non-random and informative patterns in it. The use of these methods creates a leap of faith: the measures are “validated” largely by analysis of between-individual differences but then, increasingly in the psychological therapies, are used to measure change within individuals, not just to compare scores between individuals. The belief that these are the only methods to show “validity” perpetuate the myth that idiographic information cannot be approached statistically and allows the subjectivity and individuality behind questionnaire responses to be ignored. However, the method of derangements is absolutely statistically clear and derives from mathematics that has been known since the 18th century and can be used to explore purely individual, subjective data.

We have recapped the method above, summarising: it tests whether matching of idiographic data might have happened by “luck,” i.e., down to sampling vagaries, drawing on the same statistical principles that underpin nomothetic psychometrics. The answer is that this probability is less than *p* = 0.05, i.e., statistically significant by conventional rules, if four or more correct matches are achieved. Though only four correct matches are needed for conventional statistical significance in one application of the method a 19 out of 19 matching was achieved: the probability of that the score being achieved by chance is far less than the “sigma 5,” i.e., a 1 in 3,500,000 criterion used to ascribe significance to findings in particle physics.

As with any method, the maths and probabilities are not the whole story: the method needs care to ensure that mappings are not based on spurious information. Both the Evans et al. (2002) and the Walters (2005) papers report careful steps to minimise this possibility but, as with blinding in controlled trials, this needs to be considered logically in each individual project reflecting the choice of descriptors and clear, transparent reporting of design choices.

In reviewing above the citations of the Evans et al. (2002) paper after 20 years, we understood more about why the method has been taken up so little, and looking at the original paper, particularly going back to the detail of the idiographic data, it is clear that it was written to highlight the history of the maths and one derivation of the finding. There was little focus on application of the method: this probably limited its potential impact for most psychological researchers. That it appeared in a small circulation journal before open access also meant that the method was not easily located by those for whom it had most applicability. These issues of the ecology and sociology of journals and the dissemination of methods impact all disciplines.

Another ecological or sociological issue might be that experts in qualitative data analysis may mistrust methods that seem to pull qualitative data towards what appear to be nomothetic models and epistemologies. We have some sympathy with that but hope we have demonstrated that nothing in the method diminishes that subjectivity may be at the heart of the generation of the data and at the heart of the mapping, and that the qualitative data can been shown to be communicating information validly beyond any reasonable probability that this is down to chance. We see no reason why application of such tools in any way pulls qualitative data into reductionism.

These tensions between quantitative and qualitative psychology methods rip holes in psychology’s nomological nets (Cronbach and Meehl, 1955) by neglecting quantitative exploration of individuality. One attempt to address this expanded from the ideas of Jacobson and colleagues (Jacobson et al., 1984; Christensen and Mendoza, 1986; Jacobson et al., 1986) and this forms an interesting contrast to rigorous idiography and the method of derangements.

Jacobson et al. recognised that clinicians mainly took a *per* client (and largely qualitative and narrative) approach to change in therapies and so found researchers’ focus on group aggregate change measurement largely uninteresting. They introduced two criteria to classify individuals’ score change on any single nomothetic measure across interventions as “clinically significant” and as “reliable” (hence the term “Reliable and Clinically Significant Change, RCSC, for these approaches). This method created an important literature about the assumptions in the model (Hageman and Arrindell, 1993; Follette and Callaghan, 1996; Martinovich et al., 1996; Tingey et al., 1996a, 1996b; Bauer et al., 2004; Wise, 2004). Interestingly, the simplest approach seems to have outlived the various extensions, perhaps reflecting that there is no single best way to categorise change on a single nomothetic measure that can satisfy all that people might want the categorisation to achieve. As Wise concludes:

“The RCI and CS methodology has withstood rigorous debate and survived stronger than originally conceived. Despite methodological limitations, studying RCI and CS has moved the outcomes paradigm from studying treatment groups to studying individual change within those groups. Similarly, assessment instruments must move beyond symptom focus and evaluate individuals with respect to the complex broader domains of their functional, real-world, lives in which clinically significant change is operationalized” (Wise, 2004, p.57).

However, commentaries on the RCSC tend not to challenge the myth that psychometrics can transmute psychological data to objectivity and that only nomothetic data are “hard.” All suggested measures are of course using nomothetic measure scores to categorise individuals rather than seeking to recognise and explore individuality. Bauer et al. (2004) touch on this noting:

“The second issue concerns the usage of reliability scores. From a strict methodological perspective, it is not proper to apply reliability information to single cases. With respect to this argument, one should favor these statistics based on reliability with groups of clients when comparing the results of different studies rather than with the individual client” (Bauer et al., 2004, 68).

Intensive work from the Netherlands (Smet et al., 2019; De Smet et al., 2020; Desmet et al., 2021) involving both RCSC categorisation and qualitative interviews shows the substantial individuality in clients’ appraisals of their change in therapies. Clearly, it has been valuable for psychology and therapies to have self-report nomothetic change measures for aggregate summary analyses and RCSC methods *do* convert these into counts of individuals. These methods, no less than using uncategorised nomothetic scores, minimise or ignore subjectivity, whether that of the person being classified or categorised, or that of the person doing the classifying and categorising. They comply with the strongest reason for neglect of subjective data and for the myth that psychometrics can transmute psychological data to objectivity: the yearning for universal rules with which we opened this paper. These methods have resource economy requiring only first and last scores on a nomothetic measure but this is different from exploring the rich subjectivity and individuality of clients’ experiences. Methods of “rigorous idiography” do something different: they take purely idiographic descriptors, ones that may be very subjective then test whether it can be shown that matching them to the original data was highly unlikely to have occurred by chance.

The method of derangements, as we have noted, can be used for objective data: equations and plots in Antonelli’s exam question (Antonelli, 2019) for example. However, the real strength of the method for psychology is that data unique to individuals can be demonstrated to be validly identifiable by a judge. While this validates the data it is the complete opposite of a general rule. The method only validates the mapping for the judge or judges who did the mapping and for the *n* > 4 descriptors being mapped; there is no given generalisability just as there is none in any single observer qualitative data report.

Examples of how the method can be used in general perceptual and cognitive psychology include virtually any matching of idiographic descriptors to other data. As well as the examples given above, applications might include exploring mapping non-content vocal cues to affective state: can judges match brief descriptors—“happy,” “sad,” “angry,” “confused,” “excited,” “anxious” to heavily tonally filtered clips of talk by people in those states where the filtering removes content cues but not rhythm and some other vocal cues? Can something similar be done looking at videos of people’s body language in those states but without the face being visible? In therapy research could categories of interaction and interaction ruptures map to similarly restricted data from recordings of therapy sessions? A particular area of application could be to examine the validity of the sometimes alienating terminology of some psychotherapy formulations: “Paranoid-Schizoid,” “reaction formation” even “inauthentic.” Can judges given a minimal introduction to these concepts map formulations to segments of therapy session recordings?

The method of derangements only requires a minimum of four descriptors for sets of individual data to be matched, but it can extend to any number, though there are clearly limits on the number most raters could handle in one task. The task in Walters (2005) had 19 cognitive maps to match to the men whose data created the maps. The best number for any one exploration is both a theoretical and a practical issue. Simulation work can be used to look at the theoretical issue of the power to detect systematic matches across different numbers of targets and their similarities but for human matching work, the best choices will depend on concentration span of the person doing the matching, and on the choices of targets to match. Clearly, if the method is used with idiographic data such as Rorschach responses or House-Tree-Person drawings then the more different those individuals are from each other, and the more different their Rorschach or House-Tree-Person responses, the more likely it is that the method will show significant mapping.

There are some obvious extensions of the statistics that would look at generalisability across judges and Evans et al. (2002) does report on data from two judges and two separate occasions for each matching task. The rules of probability could be used to extend the calculation of probability for one matching task to consider overall probabilities across multiple raters and/or occasions taking the method towards exploration of generalisability. We reassure ourselves that the path from Gosset’s invention of the *t*-test to recent multilevel models and, for example, bootstrap methods took decades (see Salsburg, 2001 for a very readable account of this and more of the history of statistics).

To conclude, the method of derangements can validate, to the usual criterion of *p* < 0.05, or in principle any more stringent criterion, that something systematic is communicated from the data. However, its application to idiographic data remains the antithesis of a generalisable finding: the finding applies to that dataset and rater. We believe that psychology needs to return to valuing this sort of specificity, valuing the personal and the individual and not ignoring anything that is not general. This is not just academic: if one therapist can match development formulations to session events such as rupture-repair sequences for even particular subsets of clients, this offers a new window to understand better how therapy works not in terms of general rules but working from specific cases and one judge, but with validation of the mappings that judge uses. Likewise, if detailed descriptions of at least some forensic patients’ index offences can be mapped to the descriptions of within therapy processes that block therapeutic change or contribute to dangerous incidents in inpatient care, that may be extremely valuable, even lifesaving, although it does not create general rules applicable to all offenders. Providing rigorous idiography in circumstances that are unique and inherently non-generalisable is in itself a radical shift in approaching psychological data.

To put the method in context we have juxtaposed it against the dominant methods of cross-sectional psychometrics of self-report data to show that neither traditional psychometrics nor the method of derangements distil objectivity from subjectivity but also to show that those psychometric methods are excellent to find regularities, generalities across individuals where the method of derangements can validate purely idiographic data about individual differences. We showed that both methods work from mathematics and probability to give rigour to test the data. Opening up the debates about the boundaries of the relationship of subjectivity and objectivity, and the limitations to generalisability for nomothetic as well as idiographic methods will help improve methodologies within psychology and the psychotherapies. Psychology, and many other fields, would be improved by a more transparent approach to living and working with these challenges. We hope that the special issue, and the way that the method of derangements allows validity testing of even purely idiographic subjective data will help heal the splits and help move the debates on.

## Data availability statement

The original contributions presented in the study are included in the article/supplementary material, further inquiries can be directed to the corresponding author.

## Author contributions

CE conceptualised the method and wrote the first draft of the article. CE, JC, and CP participated in writing the article. All authors contributed to the article and approved the submitted version.

## Funding

The Dirección General de Investigación y Vinculación of the Universidad de Las Américas, Quito, Ecuador (PSI.CPE.21.03) funded payment for Open Access.

## Conflict of interest

JC is the founder of PSYCTC.com which provides commercial and charitable consultancy, training and development services to the charitable and commercial sector.

The remaining authors declare that the research was conducted in the absence of any commercial or financial relationships that could be construed as a potential conflict of interest.

## Publisher’s note

All claims expressed in this article are solely those of the authors and do not necessarily represent those of their affiliated organizations, or those of the publisher, the editors and the reviewers. Any product that may be evaluated in this article, or claim that may be made by its manufacturer, is not guaranteed or endorsed by the publisher.
